# Deletion of the Mir-106b~ 25 MicroRNA cluster attenuates atherosclerosis in Apolipoprotein E knockout mice

**DOI:** 10.1186/s12944-019-1155-8

**Published:** 2019-12-03

**Authors:** Jonathan Semo, Gil Chernin, Michael Jonas, Sara Shimoni, Jacob George

**Affiliations:** 1 Heart Center, Kaplan Medical Center and the Hebrew University School of Medicine, Rehovot, Israel; 2 Nephrology and Hypertension Department, Kaplan Medical Center and the Hebrew University School of Medicine, P.O. Box 1, 76100 Rehovot, Israel

**Keywords:** MicroRNAs, Cholesterol, Atherosclerosis

## Abstract

**Background:**

MicroRNAs are short non-coding RNAs that regulate gene expression. The aim of this study was to gain an understanding of the possible role of the miR-106b~ 25 microRNA cluster in regulating atherosclerosis in mice.

**Methods:**

MiR-106b~ 25 knockout mice were outcrossed into Apolipoprotein E (ApoE) knockout background to generate double knockout mice. At 36 weeks of age, lesion size was evaluated in the aortic sinus by oil-red-O staining.

**Results:**

Lesion size was 2-fold smaller in double KO mice in comparison to ApoE KO mice**.** In addition, collagen staining showed a trend towards a stable plaque phenotype in the double KO mice. Lipid profiling of plasma samples of double KO and ApoE KO mice using FPLC revealed over 2-fold decrease in Very low density lipoprotein (VLDL) cholesterol content and a 50% decrease in low density lipoprotein (LDL) cholesterol content in double KO mice. By using target prediction software, we have identified several possible targets for the miR-106b~ 25 cluster including the VLDL and LDL receptors. We found that upon feeding miR-106b~ 25 KO mice with high fat diet, the expression of LDL and VLDL receptors was higher than in the wild-type mice, suggesting the miR-106b~ 25 cluster regulates atherosclerosis by influencing clearance of VLDL and LDL from the plasma.

**Conclusions:**

We identified the miR-106b~ 25 cluster as a novel regulator of atherosclerosis in ApoE KO mice, presumably by regulating plasma cholesterol levels.

## Background

Cardiovascular disease is common in the general population; it affects the majority of adults over age 60 years, and is still a leading cause of death worldwide [1]. Atherosclerosis is the principal determinant of cardiovascular outcome [2]. The etiology of atherosclerosis is multifactorial and involves dyslipidemia, inflammation and abnormal angio/vasculogenesis.

MicroRNAs are short non-coding RNAs that regulate gene expression by base-paring with the 3′ UTRs of target mRNAs [3]. Several microRNAs were shown to regulate atherosclerosis [4–7]. The miR-106b~ 25 cluster consists of three mature microRNAs: miR-106b, miR-93 and miR-25, and is a paralogue of the miR-17 ∼ 92 and miR-106a ∼ 363 clusters [8]. Ablation of miR-106b ∼ 25 or miR-106a ∼ 363 has no obvious phenotypic consequences, although mutant embryos lacking both miR-106b ∼ 25 and miR-17 ∼ 92 die at midgestation [9]. The miR-106b ∼ 25 cluster was reported to function as an oncogene by targeting P21 and BIM [10, 11]. In addition, this cluster was shown to regulate neural stem cell fate [12]; and both miR-106b and miR-93 were shown to impair cholesterol efflux [13, 14]. Moreover, miR-106b was shown to exert an anti-angiogenic effect in endothelial cells by inhibiting the STAT3-involved signaling pathway, via direct targeting of STAT3 [15]. Migration of thrombospondin-of 1 (TSP-1), a matricellular glycoprotein that induces vascular smooth muscle cells, (VSMCs) downregulated all three miRs of the miR-106b ∼ 25 cluster in TSP-1 treated VSMCs [16].

MiRNAs may also serve as potential diagnostic or prognostic markers in a range of disease states. In the miR-106b~ 25 cluster, increased plasma levels of miR93-5p were found to be a strong predictor of stable coronary artery disease [17]. On the other hand, mir-106b was not differentially expressed in the plasma of CAD patients compared with healthy controls [18].

We previously demonstrated the importance of the miR-106b~ 25 cluster in post-ischemic neovascularization in mice [19]. The aim of this study was to elucidate the role of this microRNA cluster in the regulation of atherosclerosis in mice.

## Methods

To obtain apoE and miR-106b~ 25 double knockout (KO) mice, miR-106b~ 25 KO mice were backcrossed for 5 generations into c57B6 genetic background, followed by crossing with apoE KO mice to obtain homozygous mice. ApoE KO littermates served as controls.

For gene expression experiments, miR-106b~ 25 wild-type or KO mice were fed with standard chow or a high fat atherogenic diet (“Western diet”: total fat of 21% by weight, 0.2% cholesterol, Harlan laboratories, Rehovot, Israel) for 3 weeks, followed by RNA isolation from the spleen and liver.

### Atherosclerosis and plaque stability in mice

Wild type mice do not develop atherosclerosis [20]. Therefore, the in-vivo model to assess the effect of miR-106b~ 25 on atherosclerosis was based on the background of ApoE KO mice. Male ApoE KO or double KO mice were fed standard chow for 36 weeks. Atherosclerotic lesions were quantified by calculating the lesion size in the aortic sinus. The heart and upper section of the aorta were removed from the animals, and the peripheral fat was carefully cleaned. The upper section was embedded in an optimal cutting temperature compound and frozen in liquid nitrogen. Other sections (10 μm thick) along the aortic sinus (400 μm) were taken for analysis. The extent of atherosclerosis was evaluated at the level of the aortic sinus. The tissue was processed and stained with Oil red O according to Paigen et al. [21]. Lesion area was determined by quantitative morphometry using the Nikon Instruments elements software. The fibrous cap area of the total plaque area was determined by Masson trichrome staining.

### Analysis of the plasma lipoprotein profile using fast protein liquid chromatography (FPLC)

Blood was drawn from ApoE KO or double KO mice after an overnight fast and collected into chilled paraoxon – coated capillary tubes to prevent in vitro lipolysis. Lipoproteins were separated by high resolution size exclusion FPLC using a Superose 6 column (Amersham Pharmacia Biotech AB, Piscataway, NJ). Aliquots of 100 μl from each 0.5 ml fraction were used for cholesterol and TG measurements.

### Real-time PCR

For analysis of microRNA in endothelial cells, RNA was isolated from H5V murine endothelial cells after treatment with ox-LDL (50 μg/ml) for 1 h, 6 h or 24 h. Total RNA was extracted with a phenol/chloroform EZRNA kit (Biological Industries, Beit Ha-Emek, Israel). For miRNA detection, 25 ng of total RNA was transcribed to cDNA using the universal RT LNATM cDNA Synthesis Kit (Exiqon, Vedbaek, Denmark). Quantitative real-time PCR was performed with Sybr Green and specific locked nucleic acid primers for miR-106b, miR-93 and miR-25 (Exiqon, Denmark), using a StepOnePlus instrument (Applied Biosystems). Results were derived by the Comparative CT (ΔΔCt) method and were normalized to the expression of U6.

### In-vivo migration assay

Eight-week old miR-106b~ 25 wild type or KO mice were injected with 3 ml of thioglycollate. After 72 h, mice were sacrificed and cells were collected by washing the peritoneal cavity with phosphate buffer saline (PBS). Cells were counted for each mouse separately.

### Flow cytometry

Total splenocytes were incubated with anti PE-CD4, anti-APC-CD25 and anti-FITC-FoxP3 antibodies (eBioscience, San Diego, CA) or their corresponding isotype controls, for 30 min at 4 °C in the dark. Cells were analyzed in a flow cytometer (BD Biosciences FACSCanto II). The results were analyzed by FACSDiva software (all from Becton Dickinson, Franklin Lakes, NJ).

### Differentiation of spleen cells to Th1 or Th2

Spleens from miR-106b~ 25 wild type or KO mice were mechanically minced and plated into 6-well tissue culture plates. Forty-eight hours after plating, non-adherent cells were transferred to fresh plates and were differentiated into Th1 (IL-12 20 ng/ml) or Th2 (IL-4 20 ng/ml) for 24 h. Cells were maintained at 37 °C, 5% CO2 in RPMI supplemented with 10% fetal calf serum, 1% penicillin/streptomycin and 1% glutamine (all from Biological Industries).

### Very low-density lipoprotein (VLDL) in-vivo clearance assay

Human VLDL (BTI Biomedical Technologies) was labeled with 50 μg of FITC dissolved in DMSO (1 mg/ml) (Sigma-Aldrich) for 2 h at room temperature. Next, conjugated VLDL was separated from free fluorochrome by gel filtration using PD10 column (GE Healthcare). We injected 100 μg of FITC-VLDL to the tail vein of ApoE- and double KO mice. Blood samples were collected into EDTA-coated tubes 1 and 15 min after injection. Plasma samples were diluted 1:20 in PBS and fluorescence was read using a BioTek synergy HT fluorescent plate reader. For each mouse, data were normalized to fluorescent intensity measured at 1 min.

### Polarization of spleen macrophages to M1 or M2 phenotype

Spleens from miR-106b~ 25 wild type or KO mice were mechanically minced and plated into 6-well tissue culture plates. Forty-eight hours after plating, non-adherent cells were aspirated and the remaining cells were subjected to m1 (IFNƔ 10 ng/ml and LPS 100 ng/ml) or m2 (IL-4 20 ng/ml) polarization. RNA was isolated from treated cells after 24 h.

### Mouse cytokine array

Cytokine array (R&D systems) was performed on growth medium collected from spleen macrophages treated with oxidized LDL for 24 h according to manufacturer’s instructions.

### Adhesion assay

Spleen macrophages were isolated from miR-106b~ 25 wild type or KO mice and treated with oxidized LDL (Biomedical Technologies, USA) 20 μg/ml or 50 μg/ml for 24 h. Cells were then plated on 96 well tissue culture plates pre-coated with fibronectin. Non-adherent cells were washed after 30 min and adherent cells were quantified with XTT cell proliferation kit (Biological Industries, Israel).

### Target prediction of miRNAs

Potential targets of miR-25, miR-93 and miR-106 were predicted using two well-established software, which are described in detail for TargetScan [22] and for RNA22 [23].

### Statistical analysis

Groups were compared using Student’s two-tailed t-test or one-way ANOVA using Graphpad Prism 5 software. Significance was set at P<0.05 (*P<0.05; **P<0.005). Results are expressed as means±SEM.

## Results

**Fig. 1:**
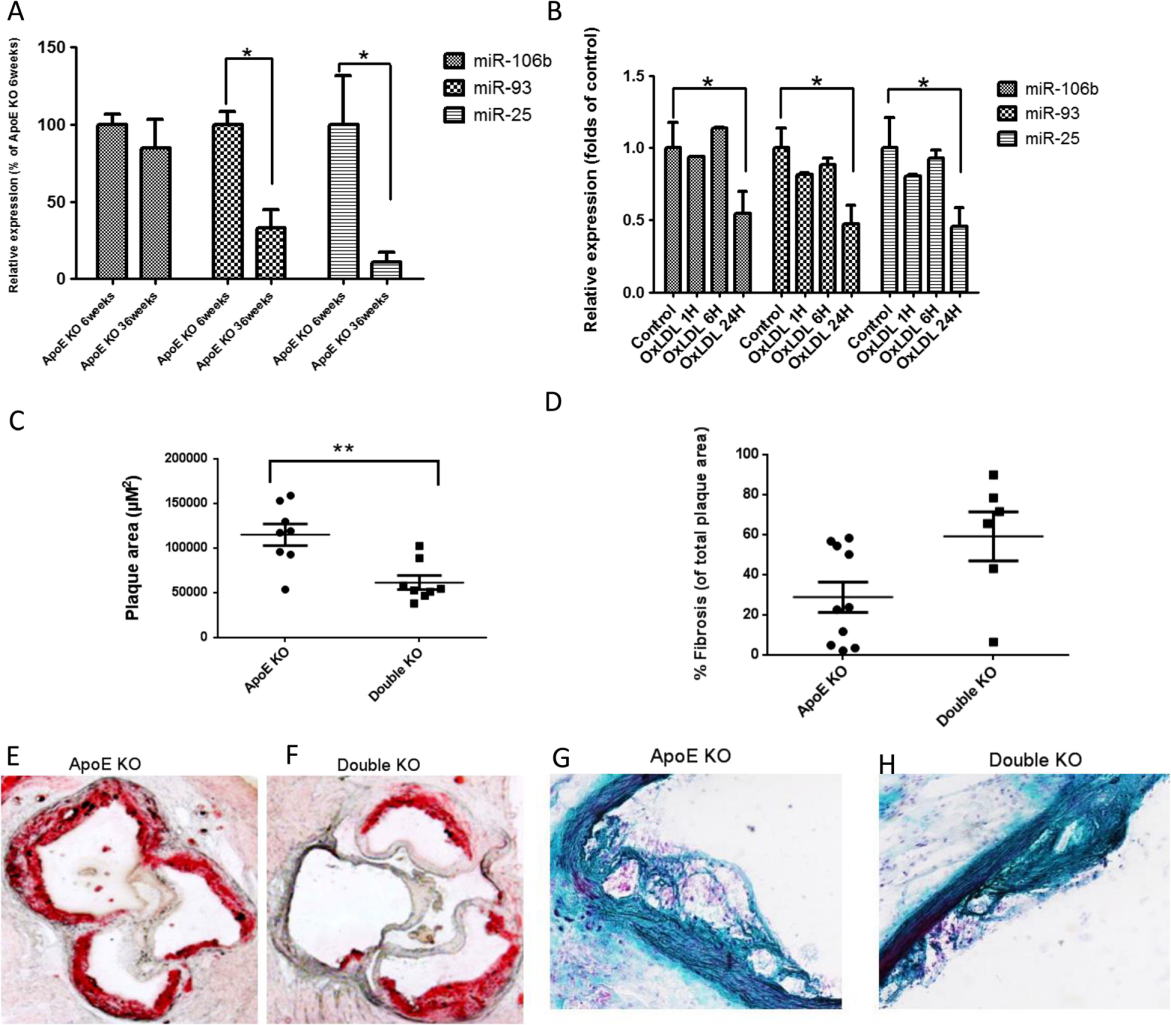
Expression and effect of miR-106b~ 25 cluster members in atherosclerosis progression (**a**) Relative expression of the miR-106b~ 25 cluster members determined by quantitative real-time PCR in aortas of 6 week or 36 week old ApoE KO mice (*n* = 8). **b** Relative expression of the miR-106b~ 25 cluster members determined by quantitative real-time PCR in murine endothelial cells subjected to oxidized (Ox) LDL treatment (20 μg/ml) for 1 h, 6 h or 24 h (*, *P* < 0.05). Relative expression was determined by qRT–PCR with specific primers for miR-25, miR-106b and miR-93. The results were derived by the DDCt method and were normalized to the expression of U6. **c** Plaque area as determined in the aortic sinus of ApoE KO (*n* = 8) or double KO mice (*n* = 8) aged 36 weeks. (**, *P* < 0.005). Representative Oil-red-O staining images of atherosclerotic plaques in the aortic sinus of (**d**) ApoE KO mice and (**e**) Double KO mice. **f** Fibrotic cap coverage of atherosclerotic plaques determined by Masson-trichrome staining. Representative images of (**g**) ApoE KO and (**h**) Double KO mice

To examine possible changes in expression of the members of the miR-106b~ 25 cluster with the progression of atherosclerosis, we used the ApoE KO model. In ApoE KO mice, expression of miR-93 and miR-25, but not of miR-106b, was decreased at age 36 weeks compared to age 6 weeks (Fig. 1a). In mouse endothelial cells, expression levels of miR-106b, miR-93 and miR-25 were downregulated 24 h, but not 1 or 6 h after treatment with oxidized LDL (Fig. 1b).

**Fig. 2:**
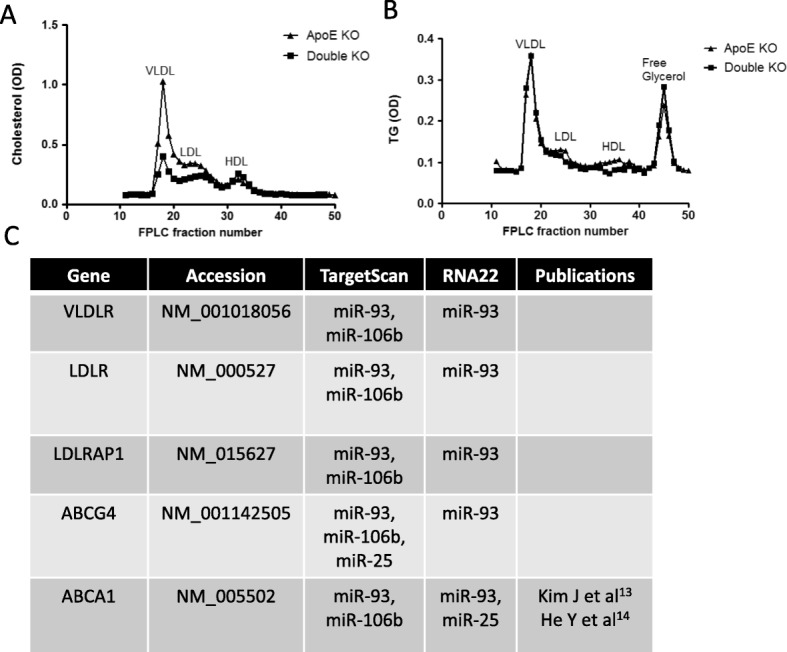
Lipid content in double KO mice (**a**) Cholesterol content in fractions corresponding to VLDL, LDL and HDL as determined by fast protein liquid chromatography (FPLC) in pooled plasma samples derived from ApoE KO or double KO mice. **b** Triglyceride content in fractions corresponding to VLDL, LDL and HDL, as determined by FPLC in pooled plasma samples derived from ApoE KO or double KO mice. **c** Table of predicted targets of members of the miR-106b~ 25 microRNA cluster according to two well-established target prediction softwares: TargetScan and RNA22

Next, we hypothesized that deletion of the miR-106b~ 25 cluster in ApoE KO mice would have a functional effect on the progression of atherosclerosis. ApoE KO and miR-106b~ 25 KO (Double KO) mice and ApoE KO littermates, which served as controls, were fed with standard chow for 36 weeks followed by evaluation of atherosclerotic lesion size and plaque stability. Lesion size was smaller in double KO mice than in ApoE KO mice, as evident by Oil-red O staining (Fig. 1e-f). In addition, collagen expression by Masson’s trichrome staining showed a trend towards a stable plaque phenotype in the double KO mice (*P* = 0.092 two-tailed t-test) (Fig. 1g-h). Lipid profiles of plasma samples of double KO and ApoE KO mice were analyzed with FPLC. Cholesterol content in the VLDL and LDL fractions, but not in the HDL fraction, was lower in double KO mice than in ApoE KO mice (Fig. 2a). There was no detectable difference in triglyceride levels (Fig. 2b).

**Fig. 3:**
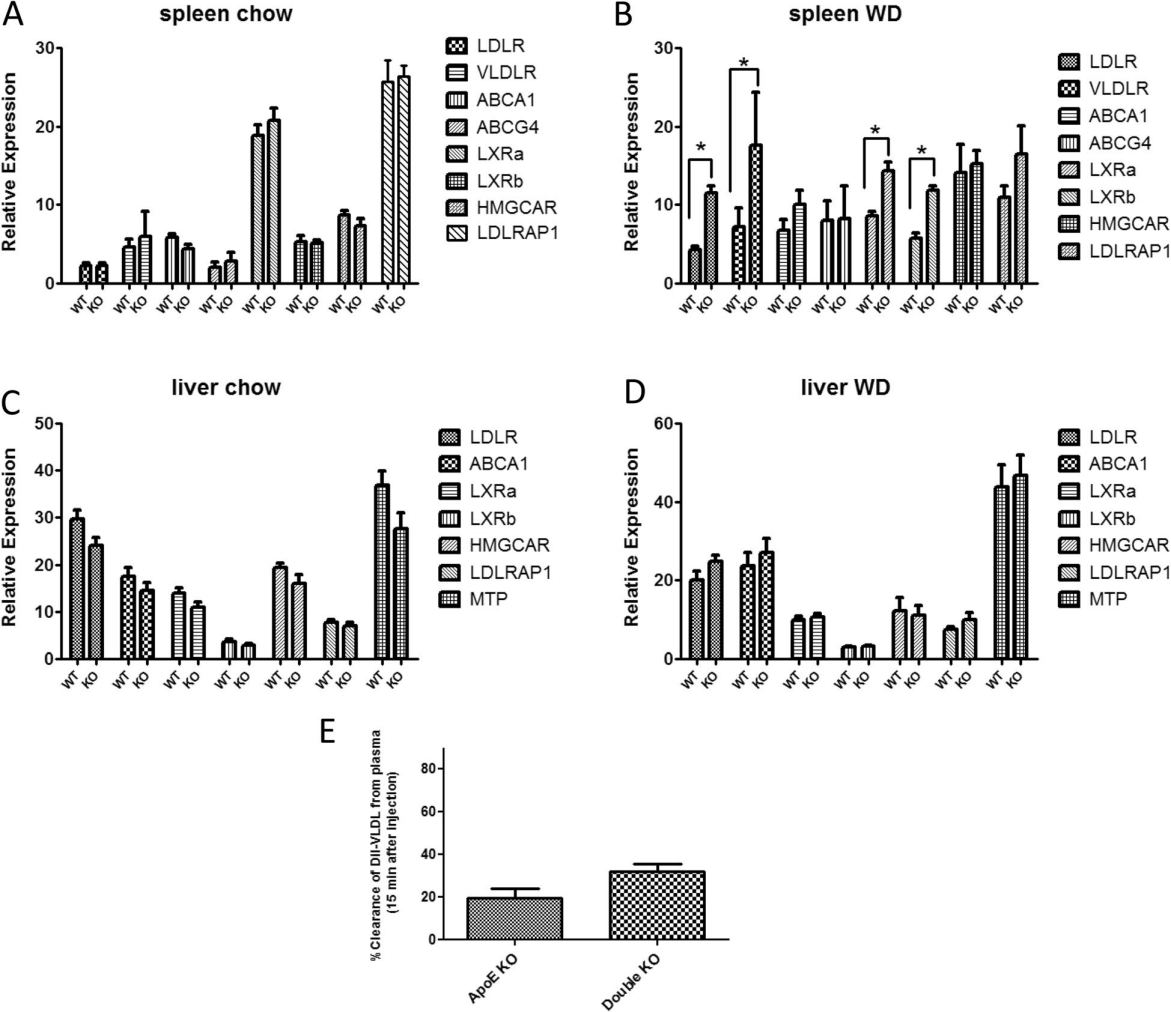
Relative expression as determined by real-time PCR of genes pertaining to cholesterol biosynthesis or uptake. Expression levels were determined in (**a**) Spleen of wild-type or 106b~ 25 KO mice fed with standard chow (*n* = 8 for each) (**b**) Spleen of wild-type or miR-106b~ 25 KO mice fed with high fat atherogenic diet for 4 weeks (*n* = 4 for each). **c** Liver of wild-type or 106b~ 25 KO mice fed with standard chow (*n* = 4 for each) (**d**) Liver of wild-type or miR-106b~ 25 KO mice fed with a high fat atherogenic diet for 4 weeks (*n* = 4 for each). (E) Clearance of FITC-VLDL from the plasma of ApoE KO mice or double KO mice 15 min following intravenous injection (100 μg/ mouse, *n* = 6 for each, *P < 0.05)

Given the pronounced reduction in VLDL and LDL cholesterol content in double KO mice, we became interested in whether the miR-106b~ 25 cluster could influence plasma cholesterol levels by targeting genes pertaining to cholesterol biosynthesis and/or transport. Figure 2c summarizes 5 genes that are targeted by members of the miR-106b~ 25 cluster according to two well-established target prediction software; the genes include the LDL/VLDL receptors and the cholesterol transporters ABCG4 and ABCA1. We analyzed expression of these 5 genes in addition to several other genes involved in cholesterol transport or metabolism in miR-106b~ 25 wild type and KO mice. Expression levels were determined in the spleen and liver of mice fed with standard chow or a high fat atherogenic diet (“Western diet”) for 3 weeks. In the spleens of mice fed standard chow, differences were not detected between miR-106b~ 25 KO and wild-type mice in the expression of genes relating to cholesterol biosynthesis or uptake (Fig. 3a). However, the expression of 4 genes: VLDLR, LDLR, LXRa and LXRb were upregulated in the spleens of miR-106b~ 25 KO mice fed a high fat diet compared to wild-type controls (Fig. 3b). No differences in gene expression were observed in the liver, regardless of the type of diet. (Fig. 3c-d).

To examine whether reduced plasma levels of VLDL in double KO mice result from increased clearance or from uptake to peripheral tissues, we injected FITC-labeled VLDL to ApoE- or double KO mice, and measured the decay of a fluorescent signal from the plasma. VLDL was more rapidly cleared from the plasma after injection to double KO than to ApoE KO mice; this difference, however, did not reach statistical significance (*P* < 0.05 *n* = 6 per group) (Fig. 3e).

**Fig. 4:**
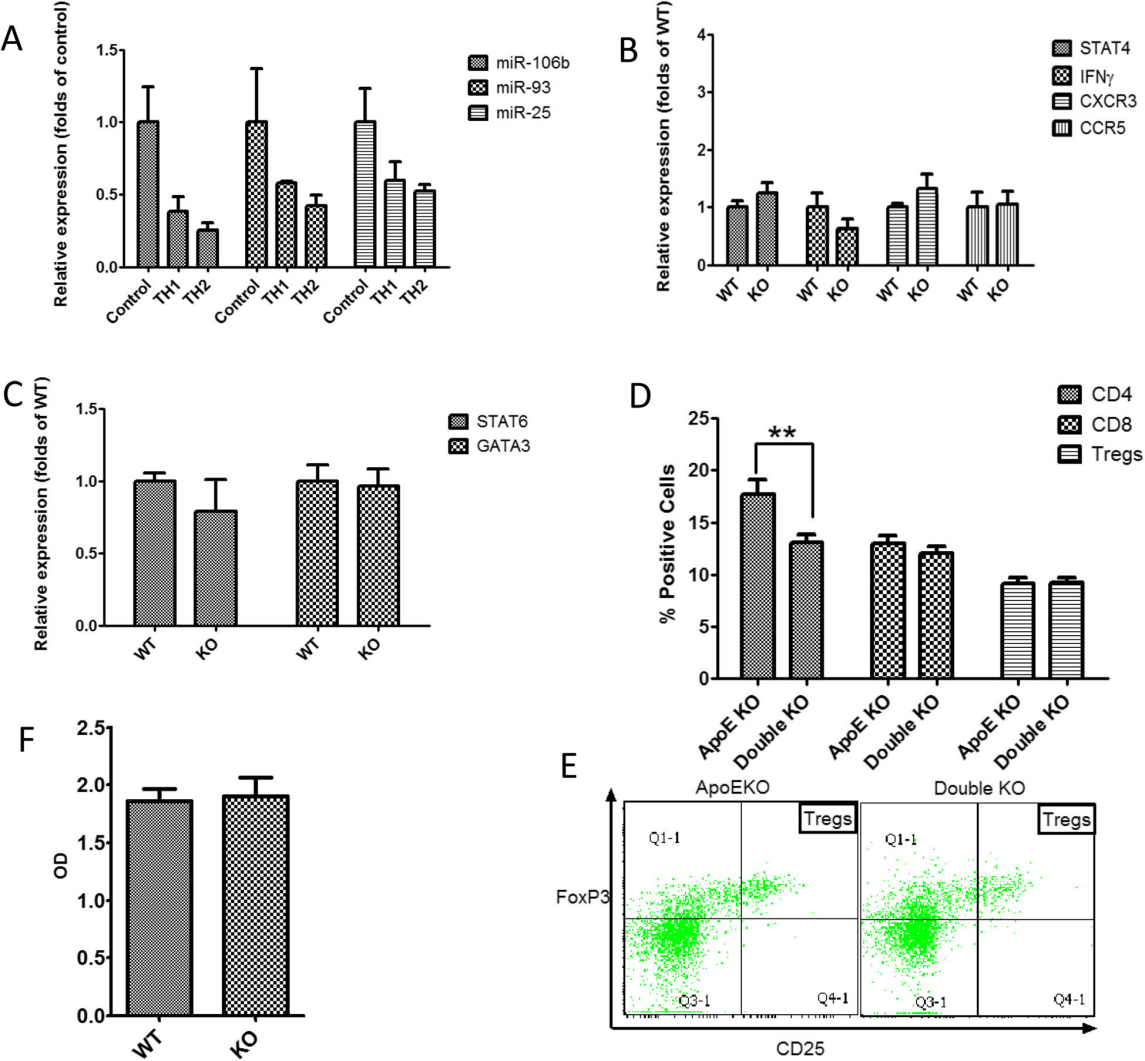
miR-106b~ 25 deletion effect on Th1/Th2 balance in mice fed a regular diet. **a** Relative expression of the miR-106b~ 25 cluster members determined by quantitative real-time PCR in spleen-derived lymphocytes subjected to Th1 (IL-12 20 ng/ml) or Th2 (IL-4 20 ng/ml) differentiation. The miR-106b~ 25 cluster members were significantly downregulated compared to undifferentiated control lymphocytes (*P < 0.05). **b-c** Relative expression of Th1 markers (**b**) or Th2 markers (**c**) determined by quantitative real-time PCR in spleen-derived lymphocytes isolated from miR-106b~ 25 wild-type or KO mice. Expression levels of all examined markers in the ApoE KO and double KO mice were not statistically different. **d** Percentage of CD4, CD8 and Treg cells (CD4posCD25highFOXP3pos) in peripheral blood cells isolated from ApoE KO or double KO mice aged 36 weeks, as determined by flow cytometry (*n* = 10 per group; **, *p* < 0.05). **e** Representative flow cytometry images of the Treg population from ApoE or double KO mice

The balance between Th1 and Th2 responses can influence the progression of atherosclerosis. Therefore, we tested the expression of miR-106b, miR-93 and miR-25 in spleen lymphocytes (of mice fed a regular diet) subjected to Th1 or Th2 differentiation. Both Th1 and Th2 differentiation resulted in significant downregulation of the miR-106b~ 25 cluster compared to undifferentiated lymphocytes (*P* < 0.05 for all cluster members). However, expression of Th1 and Th2 differentiation was similar (Fig. 4a). Moreover, when lymphocytes isolated from miR-106~ 25 wild-type or KO mice were subjected to Th1 or Th2 differentiation, expression of Th1 or Th2 related markers did not differ (Fig. 4b and c). In addition, we compared the percentage of CD4, CD8 and regulatory T cells (CD4^positive^CD25^High^FoxP3^Positive^) in the spleens of ApoE KO and double KO mice. The number of CD4 positive cells in double KO mice was lower than in ApoE KO mice after 36 weeks, but there was no difference in quantities of CD8 or regulatory T cells (Fig. 4d and e). In addition, there was no difference in anti-oxidized LDL antibody titer between the two groups (Fig. 4f).

**Fig. 5:**
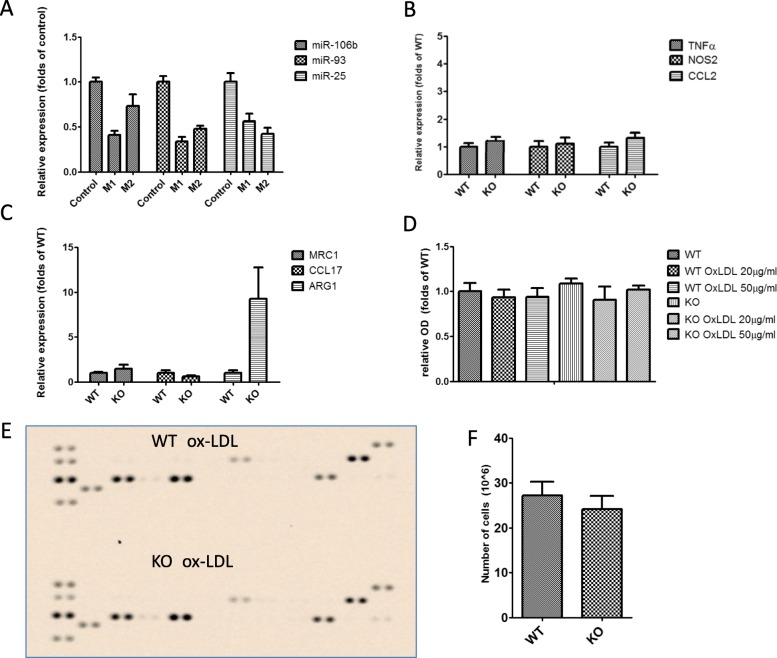
miR-106b~ 25 deletion effect on macrophages and cytokines. **a** Relative expression of the miR-106b~ 25 cluster members determined by quantitative real-time PCR in spleen-derived macrophages subjected to M1 (IFNƔ 10 ng/ml and LPS 100 ng/ml) or M2 (IL-4 20 ng/ml) differentiation. M1 and M2 polarization resulted in downregulation of the miR-106b~ 25 cluster members compared to undifferentiated macrophages (*P < 0.05). (B-C)- Relative expression of M1 markers (**b**) or M2 markers (**c**) determined by quantitative real-time PCR in spleen-derived lymphocytes isolated from miR-106b~ 25 wild-type or KO mice. **d** Relative adhesion of spleen-derived macrophages isolated from miR-106b~ 25 wild-type or KO mice and treated with oxidized LDL (20 or 50 μg/ml for 24 h). **e** Cytokine array of spleen-derived macrophages isolated from miR-106b~ 25 wild-type or KO mice and treated with oxidized LDL (20 μg/ml for 24 h)

To further explore the possible influence of miR-106b~ 25 on immune regulation in atherosclerosis, we examined whether polarization of macrophages to the M1 or M2 phenotype is of any consequence to the expression of miR-106b, miR-93 and miR-25. Both M1 and M2 polarization resulted in downregulation of the miR-106b~ 25 cluster compared to undifferentiated macrophages (*P* < 0.05). However, there was no difference in expression between the M1 and M2 phenotypes (Fig. 5a). Moreover, when macrophages isolated from the miR-106~ 25 wild-type or KO mice were subjected to M1 or M2 polarization, the expression of M1 or M2 specific markers did not differ. (Fig. 5b and c). Finally, no difference was observed in the adhesion of macrophages isolated from miR-106b~ 25 wild type or KO mice, in pro-inflammatory cytokine secretion or in-vivo migration (Fig. 5d-f).

## Discussion

Several microRNAs have been shown to influence the progression of atherosclerosis in murine models, including miR-30C [4, 7], miR-155 [6, 7] and miR-33 [5, 7]. Here, we demonstrated a novel function of the miR-106b~ 25 cluster in regulating the progression of atherosclerosis in ApoE KO mice. First, two members of the miR-106b~ 25 cluster: miR-93 and miR-25 were downregulated in ApoE KO mice that harbored advanced atherosclerotic lesions. In addition, the expression of the entire cluster in endothelial cells treated with oxidized LDL was downregulated.

We previously showed the importance of this microRNA cluster in regulating post-ischemic neovascularization [19]. Mice deficient in both ApoE and the miR-106b~ 25 cluster develop smaller atherosclerotic lesions, and this reduction in size was associated with decreased plasma levels of LDL and VLDL cholesterol. This finding could result from either decreased synthesis of cholesterol in the liver or increased cholesterol uptake by peripheral tissues. A possible explanation for the decreased plasma levels of LDL and VLDL cholesterol in the double KO mice is that blood was drawn after an overnight fast. A longer period of 24 to 48 h of food deprivation is included in most studies. MiR-106b and miR-93 were previously shown to impair cholesterol efflux by targeting the ABCA1 transporter [13, 14]. Several other proteins involved in cholesterol transport and uptake, including the LDL and VLDL receptors, are predicted to be targets of the miR-106b~ 25 cluster. We show here, that after feeding miR-106b~ 25 wild type and KO mice a high fat diet, the expression of LDL and VLDL receptors was higher in the spleen of miR-106b~ 25 KO mice. Possibly, in this setting, deletion of the miR-106b~ 25 cluster lifts its inhibitory effect on these genes, leading to their accumulation in the spleen. However, gene expression was not altered in the liver. This supports the possibility of increased peripheral uptake of cholesterol in miR-106b~ 25 KO mice, rather than reduced cholesterol production in the liver. Further support for this hypothesis comes from our observation that VLDL is more rapidly cleared from the plasma upon injection to double KO compared to ApoE KO mice. In addition, expression of LXRa was also increased in miR-106b~ 25 KO mice; LXRa gene therapy in macrophages was previously shown to reduce the size of atherosclerotic lesions in LDL-R KO mice [24].

The immune system is a dominant determinant of atherosclerosis development; and Th1 and Th2 responses may play a role in atherosclerosis progression [25, 26]. Moreover, the expression of miR-93 and miR-106b was shown to be enriched in Th1 cells compared with Th2 differentiated T cells [27]. We therefore assessed the effect of miR-106b~ 25 deletion on Th1/Th2 balance. We found no significant difference in expression of Th1 or Th2 markers in miR-106b~ 25 wild type and KO mice. The number of CD4 positive cells in the spleens of double KO mice was lower than in ApoE KO mice; this may be due to the lower atherosclerotic burden in the double KO mice. The numbers of regulatory T cells and anti-oxidized LDL antibody levels were similar between the ApoE KO and double KO mice, suggesting that miR-106b~ 25 deletion does not affect B or T cell function in-vivo. In addition, M1 pro-inflammatory macrophages are associated with atherosclerotic plaques [28, 29]. However, deletion of the miR-106b~ 25 cluster had no significant effect on polarization of macrophages, pro-inflammatory cytokine secretion, adhesion or migration, further corroborating the notion that the reduced atherosclerosis in the double KO mice is probably unrelated to immune dysregulation.

## Conclusion

In conclusion, we identified the miR-106b~ 25 cluster as a novel regulator of atherosclerosis in APOE KO mice, presumably by altering plasma cholesterol levels. The results presented here may suggest that targeting the miR-106b~ 25 cluster may have a beneficial effect on atherosclerosis progression. Further studies are needed to investigate this effect on atherosclerosis in humans.

## Data Availability

All data generated or analyzed during this study are included in this published article.

## Abbreviations

ApoE: Apolipoprotein E
LDL: Low density lipoprotein
VLDL: Very low density lipoprotein
VSMCs: Vascular smooth muscle cells
